# Prevalence of Goiter in Rural Area of Belgaum District, Karnataka

**DOI:** 10.4103/0970-0218.45373

**Published:** 2009-01

**Authors:** R Kamath, Vinod Bhat, RSP Rao, Acharya Das, Ganesh KS, Asha Kamath

**Affiliations:** Department of Community Medicine, Melaka Medical College, Malaysia; 1 Department of Community Medicine, KMC, Manipal, India; 2 Department of Community Medicine, KS Hegde Medical College, Mangalore, Karnataka, India; 3 Department of Community Medicine, Kasturba Medical College, Mangalore, India

**Keywords:** Determinants, goiter, multiple logistic regression analysis, prevalence

## Abstract

**Background:**

To determine the prevalence of goiter and to study the factors influencing goiter among people of the rural community in Karnataka state, a community based study. Setting and Study Design: A cross sectional study was carried out to find out the prevalence of goiter in a rural community of Belgaum district. The study was conducted by house-to-house survey for a period of one month.

**Materials and Methods:**

Two villages (Handiganur and Gundwad) were selected randomly from Belgaum and Raibag taluks of Belgaum district. All the family members in each household were examined for the presence of goiter using WHO criteria. Iodine content of the salt sample obtained from each household was estimated by using spot testing kits. Information regarding the determinants of goiter was collected and recorded in a pre tested proforma. Data collected was analyzed using SPSS statistical packages.

**Results:**

The prevalence of goiter among rural population was found to be 16.6%. Goiter of grade 1 was 15.7% and that of grade 2 was 0.9%. Prevalence among males and females were 7.2% and 21.8%, respectively. The prevalence of goiter was highest among adolescents. Estimation of iodine content in the salt sample revealed that 50% of samples had adequate iodine content (≥15 ppm). Multiple Logistic Regression Analysis revealed that females of the age group 10-49 years were independently associated with goiter.

**Conclusion:**

Prevalence of goiter was relatively high and therefore constituted a public health problem in this region.

## Introduction

Iodine deficiency disorder (IDD) has been recognized as a public health problem in India. Surveys conducted in various states showed that no state in the country is free from IDD. Sample surveys conducted in 25 states and 5 Union territories of the country revealed that out of 282 districts surveyed so far, IDD is a major public health problem in 241 districts where the prevalence is more than 10%. It has been estimated that in India 200 million people are living in iodine deficient areas, 71 million persons are suffering from goiter and other IDD.(1,2) Initially confined to hilly regions, new endemic areas of goiter are subsequently identified all over the country. Enlargement of thyroid gland is the common manifestation of the IDD and goiter prevalence survey is used as diagnostic tool for identifying areas of IDD. Failure to undertake early detection and intervention measure results in secondary disabling conditions.

Very few community based studies were conducted in the past to estimate the problem of IDD in India. Such studies will be very useful for developing intervention measures in iodine deficient areas. In view of the above context, the present study was conducted to study the goiter prevalence in a rural area of Belgaum district of Karnataka state.

## Materials and Methods

Community-based cross sectional study was carried out during January 2005 in Belgaum district of Karnataka State. The study was conducted as per the directives of State Goiter Cell, Karnataka. Goiter survey was conducted on a random sampling basis. First, two taluks from the district were taken randomly for the study. Samples were collected based on 1% of the taluk population from a village in each taluk that included all ages and both sexes. Thus two villages, one each from Belgaum and Raibag taluks of Belgaum district were selected by simple random technique. All the houses of these two villages were taken for the study. Eligibility criteria for inclusion in the survey were membership in the household, defined as all persons who are biologically related and eating from a common kitchen. We could meet a total of 950 subjects of all age group including males and females during the survey.

With prior permission of the District Health Officer of Belgaum district, the team visited the concerned villages along with the help of paramedical staff of the concerned primary health centers. The study was conducted by making house-to-house visits, interviewing and examining all the individuals in all the families of the village. Initially the household schedule was filled up by interrogation and then the subjects were examined for the presence of goiter and graded as per the standard WHO guidelines. Goiter assessment was done by team of experts that included four members. Proper training on assessment of goiter was given to the team by the chief investigator before starting the study so that uniformity is maintained and inter observer variation was minimized. A pilot study was conducted at the beginning to pretest the instruments and determine the feasibility of this project. Determinants of goiter such as type of diet, type of salt used, storage pattern and place of storage of salt, source of drinking water were directly observed by the investigator in the house visited and recorded in the proforma. Iodine content of salt sample of the household was estimated by using spot testing kits supplied by state goiter cell, Bangalore.

Enlargement of thyroid gland was assessed by clinical examination and the goiter was graded as follows:

**Grade 0:** Thyroid gland was neither palpable nor visible/no goiter.

**Grade 1:** A mass in the neck that is consistent with an enlarged thyroid that is palpable but not visible when the neck is in normal position. The mass moves upwards with deglutition/goiter palpable but not visible.

**Grade 2:** A swelling in the neck that is visible when the neck is in normal position and is consistent with enlarged thyroid when the neck is palpated/goiter visible and palpable.

Data regarding the determinants of goiter, clinical examination findings and the results of the spot testing of salt for iodine content were compiled and analyzed using SPSS statistical package. The results are expressed in terms of proportions and depicted in the form of tables and bar diagrams. Chi square test, multiple logistic regression analysis were performed to calculate adjusted odds ratio and its 95% confident intervals.

## Results

Out of the total 950 subjects examined, 334 subjects were males and 616 subjects were females. The overall prevalence of goiter was found to be 16.6%. Prevalence of Grade 1 was 15.7% and that of grade 2 was 0.9%.Prevalence of palpable and visible goiter was significantly high among females (21.8%) when compared to that of males (7.2%) (χ^2^ =15, *P*≤0.001).

Prevalence was found to be high among 10-19 years age group (26%) and it remains low among 0-9 years age group (4%). Prevalence is 24% among (20-29) years age group followed by 30-39, 40-49 (each 16%), 50-59 (10%) and 60 and above age group (5%). The difference found in age group was found to be significant (χ^2^=13.875, *P*=0.003). There is no significant difference in prevalence of goiter among those with various risk factors for IDD. Present study also shows that about 45% of the population had access to iodized salt and 50% of the people used salt with adequate iodine content [Table 1]. Multiple logistic regression analysis revealed that adjusted odds ratio was significantly high for the age group ranging from 10 to 49 years. Females have a higher likelihood of goiter (Odds ratio=3.6, 95% Confident Interval= 2.5-5.8). [Table 2].

**Table 1:** Prevalence of goiter according to its determinants

Determinants	Number of subjects	%	Number of individuals with goiter	Prevalence	χ^2^, *P*
Dietary habits					
Vegetarians	414	43.58	61	14.7	1.9, 0.2
Non vegetarians	536		97	18.1
Salt used					
Rock salt	515		90	17.5	0.6, 0.4
Powdered salt	435	45.8	68	15.6	
Storage pattern					
Kept open	235	24.74	31	13.2	2.7, 0.1
Kept closed	715		127	17.8
Storage					
Near the fire	532		84	15.8	0.6, 0.4
Away from fire	418	44.00	74	17.7	
Iodine content					
Adequate	475		78	16.4	1.1, 0.8
Inadequate	475	50.00	80	16.8	
Drinking water					
Tube well	762		129	16.9	0.2, 0.6
Shallow well	188	19.79	29	15.4	

**Table 2:** Multiple logistic regression analysis

Determinants	OR (Unadjusted)	95% CI	OR (adjusted)	95% CI	*P* value
Gender					
Male	1	-	-	-	-
Female	3.6	2.2-5.8	3.6	2.3-5.9	<0.001
Age group					
60 and above	1	-	-	-	0.338
1-9	0.7	0.2-2.4	2.0	0.5-8.8	<0.001
10-19	6.1	2.4-16.4	7.6	3.1-18.4	<0.001
20-29	5.3	2.1-14.5	5.9	2.4-14.6	0.004
30-39	3.8	1.4-10.8	4.0	1.6-10.3	0.003
40-49	3.9	1.4-11.7	4.5	1.7-12.2	0.14
50-59	2.2	0.6-7.7	2.4	0.8-7.5	-

## Discussion

The prevalence of goiter among general population was found to be 16.6%. When the prevalence exceeds 10% in a defined geographical area, the problem is said to be endemic. Prevalence differs from place to place and state to state in India. A study done at coastal area of Karnataka in Udupi district among 8-10 years age group showed a prevalence of 30%.(3) Gakkhar *et al*. found the prevalence as low as 2.4% among 6-15 years age group in Jabalpur.(4) Chandra *et al*. showed a prevalence of 38.8% in Kolkata.(5) Sundaram *et al*. in 1997 estimated a low prevalence of 2.5% in Belgaum district.(6) Patowary *et al*. during 1998 assessed the prevalence of goiter in Kamalpur district of Gauhati among 3990 population and found out that highest prevalence of goiter was 37.3 % in the age group of 13-18 years. There was significant increase in the prevalence rate in females (26.8%) over males (8.3%).(7) Hayath *et al*. in 1997 conducted a study in a rural population of Lucknow with a sample size of 9122 selected by multistage sampling. Prevalence of goiter was 20.3% in the population surveyed. Prevalence of goiter was higher in females (25.3%) than males (15.3%).(8) Shanker *et al*. conducted a house to house survey in Sikkim to determine the prevalence of goiter and found out that overall goiter prevalence was 54%. In the age group of 14 years, goiter prevalence was 55.3% for males and 56.5 for females. Goiter prevalence in males was 48.6% and in females was 59.6% and this difference was statistically significant.(9) But the present study shows higher prevalence in 2 taluks of the Belgaum district indicating that they are endemic areas and goiter remains as public health problem among adult population in rural Belgaum district.

The government of India launched a centrally assisted national goiter control programme in 1962. The programme was renamed as National iodine deficiency disorder contol programme (NIDDCP) in 1992. Government also took a policy decision to universal iodization of edible salt in the same year. The goal of NIDDCP was to reduce the prevalence of IDD below 10% by 2010.(10) The present survey conducted after 10 years of implementation of universal iodization of edible salts did not show much impact on the prevalence of goiter in rural areas of Belgaum district. However, the present study shows that about 45% of the population had access to iodized salt and 50% of the salt samples tested showed adequate iodine content (15 PPM at consumer level).

The prevalence of goiter found in the present study was high among adolescents because of physiological demand. High prevalence among females was attributable to continued demand for pregnancy and childbirth. Hence females and individuals of 10-49 years of age were independently associated with high prevalence of goiter. However, this depends on the age of onset of goiter. Half the people of the region consumed iodized salt with adequate iodine. Determinants of goiter such as type of diet, type of salt, storage pattern, and place of salt storage and drinking water source were not found to be significantly affecting the prevalence in the present study. This may be because of their iodine requirement being met by food or water. Environmental factors other than iodine deficiency may also have a possible role for the prevalence of endemic goiter in the region.

Urinary iodine excretion level could not be estimated in the present study. Drinking water and food samples could not be tested for iodine content. Also we could not examine the subjects who were not available in the village during our field visits. Thus, it is concluded that goiter is a major public health problem in this region. Further investigation becomes necessary to arrive at definite cause of high prevalence of goiter in this population.
